# Multidisciplinary predialysis education reduces incidence of peritonitis and subsequent death in peritoneal dialysis patients: 5-year cohort study

**DOI:** 10.1371/journal.pone.0202781

**Published:** 2018-08-23

**Authors:** Cheng-Kai Hsu, Chin-Chan Lee, Yih-Ting Chen, Ming-Kuo Ting, Chiao-Yin Sun, Chun-Yu Chen, Heng-Jung Hsu, Yung-Chang Chen, I-Wen Wu

**Affiliations:** 1 Department of Nephrology, Chang Gung Memorial Hospital, Keelung, Taiwan; 2 Division of Endocrinology and Metabolism, Chang Gung Memorial Hospital, Keelung, Taiwan; 3 College of Medicine, Chang Gung University, Taoyuan, Taiwan; International University of Health and Welfare, School of Medicine, JAPAN

## Abstract

**Background:**

Technique failure secondary to peritonitis is a grave impediment to remain in peritoneal dialysis (PD) therapy leading to high mortality. Multidisciplinary predialysis education (MPE) has shown improvement in outcomes of chronic kidney disease (CKD) patients. However, the legacy effects of MPE in PD patients remain unclear.

**Methods:**

All patients who started PD at single hospital in 2007–16 were enrolled. The incidences of peritonitis and peritonitis-related mortality were compared between MPE recipients and non-recipients. The content of the MPE was standardized in accordance with the NKF/DOQI guidelines. Kaplan-Meier analysis and Cox proportional hazards model were applied to identify the prognostic factors associated with peritonitis-free survival.

**Results:**

Of 398 PD patients, 169 patients had received MPE before starting PD. The patients of MPE group had a lower peritonitis rate [median (IQR) 0 (0.29) versus 0.11 (0.69) episodes/person-year, *P*< 0.001] and a lower percentage of peritonitis-related deaths (3.6% versus 8.7%, *P* = 0.04) compared with the non-MPE group. The median time to the first episode of peritonitis in the non-MPE and MPE groups was 33.9 months and 46.7 months, respectively (Cox-Mantel log rank test, *P* = 0.003). Cox regression analysis revealed that MPE assignment (HR: 0.594; 95% CI: 0.434–0.813, *P*< 0.001) were significant independent predictors for peritonitis-free survival.

**Conclusions:**

An efficient standardized MPE program may prolong the time to the first episode of peritonitis and reduce peritonitis rate, independent of age, gender, diabetes, hypertension, educational status and PD modality. Subsequently, decreased peritonitis-related death.

## Introduction

The incidence of end-stage renal disease (ESRD) is increasing worldwide [1]. The prevalence of ESRD was 3,138 per million in Taiwan, 2,411 per million in Japan and 2,043 per million in the United States, respectively, in 2013 [1]. The hemodialysis (HD) is the most common renal replacement modality in most countries, including Taiwan. However, peritoneal dialysis (PD) is a home-based and self-care treatment for ESRD and has been the predominant dialysis modality in other countries, such as Hong Kong, representing the 76 percent of their ESRD population [1]. The PD preserves residual renal function, could lead to efficient clearance of median-sized molecules such as β2-microglobulin and better quality of life than HD [2]. In addition, PD is associated with lower risks of hepatitis C viral infection [3]. For all these reasons, the ideal of “PD-first” has been called growing attention in the renal communities. Technique failure secondary to peritonitis is a grave impediment to remain in PD therapy leading to high mortality [4, 5]. A short interval from PD initiation to the first episode of peritonitis is also associated with poorer outcomes [6, 7]. Optimal and efficient strategies to prevent occurrence of peritonitis and prolong the time from PD initiation to the first episode of peritonitis remain crucial to improve patient outcome.

Previous studies showed that Multidisciplinary predialysis education (MPE) decreased the incidence of dialysis and reduced mortality in late-stage chronic kidney disease (CKD) patients [8]. In addition, MPE was associated with better clinical outcomes in terms of urgent dialysis and cardiovascular events among CKD patients [9]. Through acquisition of knowledge on self-management, MPE could slow renal progression and reduce hospitalization events in late-stage CKD patients [10]. Moreover, MPE has legacy effect on dialysis period in the medical cost of the first 6 months of hemodialysis patients, because of reduction on the inpatient and total medical expenditures. The reduction has been attributed to decreased inpatient service utilization from cardiovascular causes and vascular access-related surgeries [11]. The beneficial effect of MPE on PD patients were less discussed. Whether the MPE could exert any protective effect on the occurrence of peritonitis from the empowerment of self-care technique remains unclear.

In this prospective cohort study, we aim to investigate the impact of MPE on the occurrence of peritonitis, time to first episode of peritonitis and patient outcomes of PD patients who receive this educational program in accordance with the guidelines of the National Kidney Foundation Dialysis Outcomes Quality Initiative (NKF/DOQI) [12]. We hypothesized that patients who receiving MPE in their predialysis period could have less episodes of peritonitis, longer time to the first-peritonitis and lower peritonitis-related mortality, than those patients who did not receive MPE.

## Materials and methods

### Patient characteristics and settings

All patients who starting PD at Department of Nephrology, Chang Gung Memorial Hospital, Keelung, from July 1, 2007 to December 31, 2016 were prospectively follow-up for 5 years from PD initiation. Patients were divided into MPE group and non-MPE group according to whether the subjects had ever received MPE before starting renal replacement therapy. Patients with previous treatment of maintenance hemodialysis, recovery of renal function withdrawing dialysis or recipients of renal transplants were excluded. The study endpoints, including episodes of peritonitis and outcomes after peritonitis (including hospitalization, technique failure, switching of modality into hemodialysis or death) were accurately recorded. Patients who drop-out from PD (death, renal transplant, switch to hemodialysis) before development of first peritonitis were censored. This study was conducted in adherence to the Declaration of Helsinki and approved by the Institutional Review Board at Chang Gung Memorial Hospital (IRB-96-0408B, 100-0040A3, 102-0573C, 103-5130C). The informed consent was obtained from all patients.

Information was collected for further analyses, including demographic variables, comorbid conditions upon initiation of peritoneal dialysis (diabetes, hypertension, gout, coronary artery disease, heart failure, cerebrovascular accident, peripheral arterial occlusive disease, lung disease, liver cirrhosis), educational status and obvious uremic symptoms on dialysis initiation (vomiting, fluid overload, hyperkalemia, encephalopathy or uremic bleeding). In addition, biochemistry, nutritional and dialysis related parameters were assessed upon initiation of PD. These parameters included serum urea nitrogen, creatinine, estimated glomerular filtration rate, sodium, potassium, calcium, phosphate, hemoglobin, albumin, total cholesterol, normalized protein catabolic rate (nPCR); peritoneal, renal and total Kt/V urea and creatinine clearance (CCr).

Diagnosis of peritonitis was made when at least 2 of the following criteria were present: (1) clinical features consistent with peritonitis, such as abdominal pain and/or cloudy dialysis effluent; (2) white cell count of dialysis effluent > 100/μL (after a dwell time of at least 2 hours), with > 50% polymorphonuclear cells; and (3) positive dialysis effluent microbial culture report [13]. The episodes of peritonitis and responsible microorganisms were defined for each patient.

### MPE and customary care

The concise methods of our MPE program have been described in previous reports [8, 10]. Briefly, the MPE team comprised a nurse of case management, social workers, dietitians, 10 nephrologists, and HD and PD patient volunteers. The program included delivery of knowledge on nutrition supplement, lifestyle modification, nephrotoxin avoidance, dietary principles and pharmacological regimens by case-management nurse in periodical fashion, according to their CKD stage by NKF/DOQI guidelines. Monitoring of CKD complications, preparation for timely initiation of RRT, care of vascular or peritoneal access, and registration for inclusion in the renal transplant waiting list were also instructed for late stage CKD patients. Shared decision making was performed for these patients for their choice of renal replacement modality selection. The benefit, disadvantage and self-care for different modality was explained. All patients also received dietary counseling biannually from a dietitian. The MPE program was discontinued once the patients initiate dialysis therapy. In contrast, the non-MPE group of patients received customary care from the same group of nephrologists, who instructed patients regarding the renal function, evaluation of laboratory data and the clinical indicators of renal failure as well as treatment strategies. Writing materials or booklets were given to patients if they were difficult to understanding verbal instructions without help of case-management nurse [8, 10].

### Statistical analysis

Descriptive statistics were expressed as means ± standard deviation (SD) or median (interquartile range, IQR). Discrete variables were presented as frequency and percentage. Kolmogorov-Simirnov method was used to test normality of numerical variables. Differences in continuous variables between the 2 groups were analyzed by Student t-test or Mann Whitney U test. Differences in categorical variables between the 2 groups were compared by chi-square test or Fisher’s exact test. Analysis of peritonitis-free survival was derived from the Kaplan-Meier analysis. The Cox proportional hazards model was applied to identify the prognostic factors associated with peritonitis-free survival. Statistical analysis was performed with the Statistical Package for the Social Sciences (SPSS) version 21.0 (SPSS, Inc., USA). All statistical tests were two-tailed, and a *P*-value< 0.05 was considered statistically significant.

## Results

**Fig 1:**
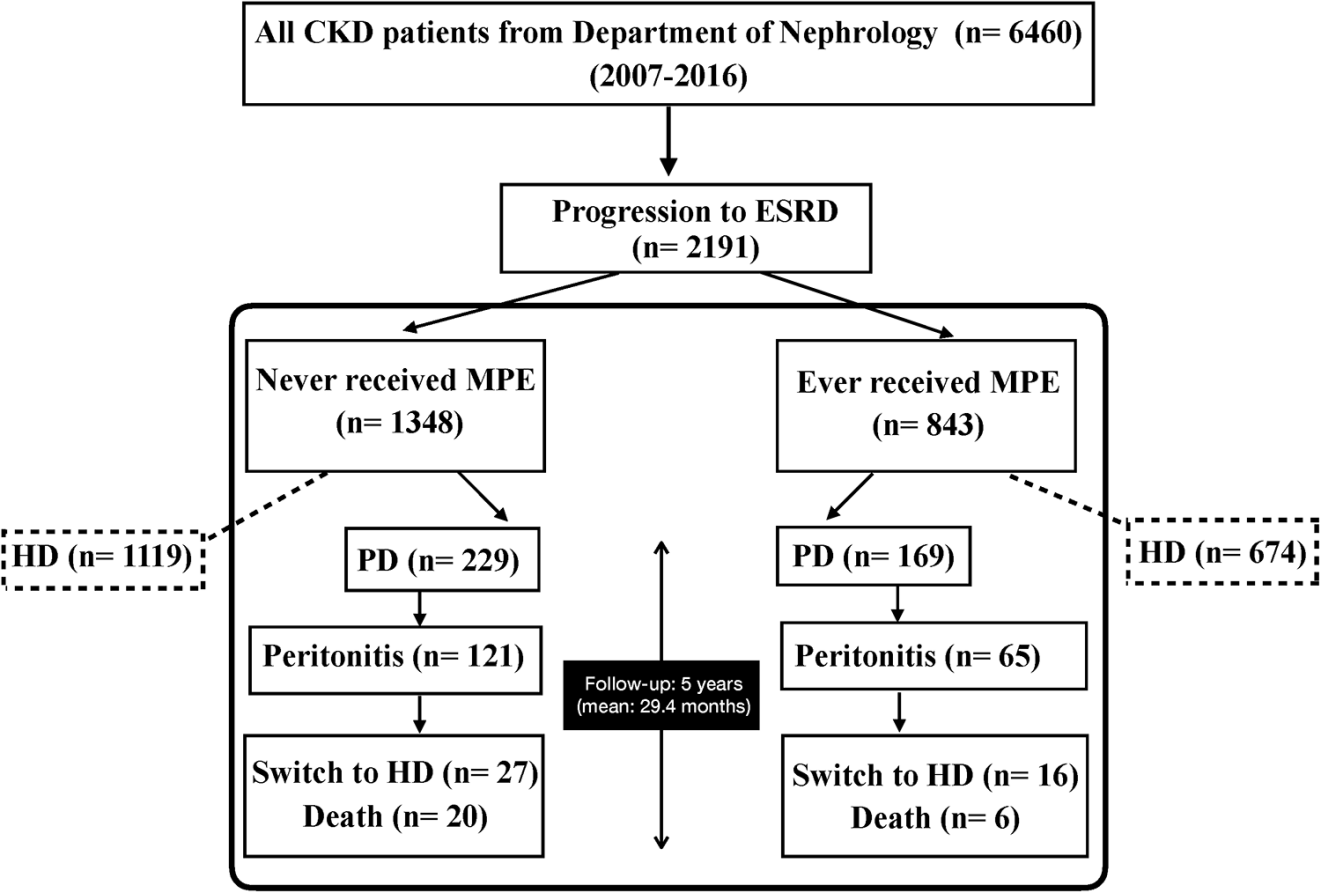
Enrollment flowchart and patient status. Abbreviation: CKD, chronic kidney disease; ESRD, end stage renal disease; MPE, multidisciplinary predialysis education; PD, peritoneal dialysis; HD, hemodialysis.

Among all 6460 CKD patients of Department of Nephrology, 2191 progressed to ESRD from July 1, 2007, to December 31, 2016. From them, 843 patients had received MPE (median duration of MPE intervention was 22 months, IQR: 7.3–39.8 months) and 169 patients (20%) had subsequently opted PD as long-term renal replacement modality. On the other hand, among 1348 non-MPE patients, 229 patients (17%) initiated PD (Fig 1). From all these patients, the mean age was 60.4 ± 16.4 years old and 185 (46.5%) of them were men. The Table 1 summarizes the baseline characteristics of patients at starting of PD. MPE recipients were older (63.1 ± 16.2 versus 58.5 ± 16.4 years old, *P* = 0.006), were less likely to be man (39.1% versus 52%, *P* = 0.01) but had higher prevalence of diabetes (60.4% versus 43.7%, *P*< 0.001) than the non-MPE recipients. The MPE group had lower baseline educational levels (*P*< 0.001) and were more likely to use automated PD (APD) than patients of non-MPE group (49.7% versus 39.7%, *P* = 0.05). There were no differences in initial laboratory findings, baseline peritoneal equilibration test (PET) and PD adequacy between two groups of patients.

**Table 1:** Demographic and clinical characteristics of patients at initiation of peritoneal dialysis.

	All patients (n = 398)	Non-MPE (n = 229)	MPE (n = 169)	*P*-value
**Age (years)**	60.44 ± 16.44	58.5 ± 16.39	63.08 ± 16.19	0.006
**Male, No. (%)**	185 (46.5)	119 (52.0)	66 (39.1)	0.01
**Primary disease**				
Diabetes, No. (%)	202 (50.8)	100 (43.7)	102 (60.4)	0.001
HTN, No. (%)	338 (84.9)	192 (83.8)	146 (86.4)	0.5
Gout, No. (%)	124 (31.2)	63 (27.5)	61 (36.1)	0.07
CAD, No. (%)	55 (13.8)	30 (13.1)	25 (14.8)	0.6
HF, No. (%)	54 (13.6)	31 (13.5)	23 (13.6)	0.9
CVA, No (%)	49 (12.3)	31 (13.5)	18 (10.7)	0.4
PAOD, No. (%)	14 (3.5%)	8 (3.5)	6 (3.6)	0.9
Lung disease, No. (%)	27 (6.8)	17 (7.4)	10 (5.9)	0.6
Liver cirrhosis, No. (%)	9 (2.3)	6 (2.6)	3 (1.8)	0.6
**Initial labroratory findings**				
BUN (mEq/L)	121 ± 46	120 ± 49	122 ± 42	0.8
Cr (mEq/L)	12 ± 6.5	12 ± 4.8	13 ± 8.1	0.1
Na (mEq/L)	135.8 ± 7.7	136.2 ± 4.1	135.5 ± 10.7	0.4
K (mEq/L)	3.95 ± 1.09	3.92 ± 0.75	3.99 ± 1.43	0.6
Ca (mg/dL)	9.17 ± 1.03	9.16 ± 1.05	9.19 ± 1.00	0.8
P (mg/dL)	4.97 ± 1.44	4.94 ± 1.49	5.00 ± 1.38	0.7
Hemoglobulin (g/dL)	10.47 ± 1.43	10.52 ± 1.48	10.41 ± 1.36	0.4
Albumin (g/dL)	3.5 ± 0.61	3.50 ± 0.64	3.49 ± 0.56	0.9
Cholesterol (mg/dL)	195.1 ± 53.8	194.9 ± 59.2	195.5 ± 45.99	0.9
**Educational status, No. (%)**				<0.001
Below elementary	79 (19.8)	41 (17.9)	38 (22.5)	
Elementary	111 (27.8)	52 (22.7)	59 (34.9)	
Junior high school	52 (13)	33 (14.4)	19 (11.2)	
Senior high school	96 (24.1)	63 (27.5)	33 (19.5)	
University	60 (15.0)	40 (17.5)	20 (11.8)	
**Baseline PET and PD adequacy**				
Peritoneal Kt/V urea	1.59 ± 0.51	1.60 ± 0.55	1.57 ± 0.45	0.6
Renal Kt/V urea	0.62 ± 0.54	0.63 ± 0.59	0.61 ± 0.46	0.7
Total Kt/V urea	2.19 ± 0.54	2.22 ± .055	2.16 ± 0.59	0.3
Peritoneal CCr (L/week)	39.93 ± 15.52	41.01 ± 17.37	38.49 ± 12.55	0.1
Renal CCr (L.week)	35.61 ± 33.73	36.79 ± 37.44	34.04 ± 28.08	0.4
Total CCr (L/week)	74.27 ± 32.60	76.91 ± 34.74	70.99 ± 29.27	0.08
nPCR (g/kg/day)	1.06 ± 0.48	1.06 ± 0.32	1.05 ± 0.63	0.7
**Peritoneal modalities**				0.05
CAPD, No. (%)	223 (56)	138 (60.3)	85 (50.3)	
APD, No. (%)	175 (44)	91 (39.7)	84 (49.7)	

**Abbreviations**: MPE, multidisciplinary predialysis education; HTN, hypertension; CAD, coronary artery disease; HF, heart failure; CVA, cerebrovascular accident; PAOD, peripheral arterial occlusive disease; BUN, blood urea nitrogen; Cr, creatinine, serum; eGRF, estimated glomerular filtration rate PET. peritoneal equilibration test; CCr, creatinine clearance; nPCR, normalized protein catabloic rate; CAPD, continuous ambulatory peritoneal dialysis; APD, automated peritoneal dialysis.

Table 2 shows clinical outcomes of peritonitis between the two groups. After a 5-years of follow-up (mean follow-up duration: 29.4 months; 30.1 months in MPE group vs. 28.5 months in non-MPE group) the MPE patients had significantly less peritonitis [0.29 ± 0.72 vs. 0.64 ± 1.5 episodes/person-year or median (IQR): 0 (0.29) vs. 0.11 (0.69) episodes/person-year, *P*< 0.001] than non-MPE patients. MPE group had lower peritonitis-related death rates compared to non-MPE group (3.6% versus 8.7%, *P* = 0.04). However, the frequency of hospitalization and the percentage of technique failures requiring shifting of modality to HD (due to either peritonitis or poor fluid management) did not differ significantly between two groups (Table 2).

**Table 2:** Clinical outcomes between the Non-MPE group and the MPE group.

	Non-MPE (n = 229)	MPE (n = 169)	*P*-value
Incidence of peritonitis, median (IQR), episodes/person-year	0.11 (0.69)	0 (0.29)	<0.001*
Frequency of hospitalization, median (IQR), episodes/person-year	1.15 (2.04)	1.36 (2.43)	0.66*
Shift to hemodialysis due to peritonitis, No (%)	27 (11.8)	16 (9.5)	0.46
Shift to hemodialysis due to poor fluid management, No (%)	5 (2.2)	3 (1.8)	0.184
Deaths due to peritonitis, No (%)	20 (8.7)	6 (3.6)	0.04

*p-value using Mann-Whitney U test.

IQR, interquartile range

**Fig 2:**
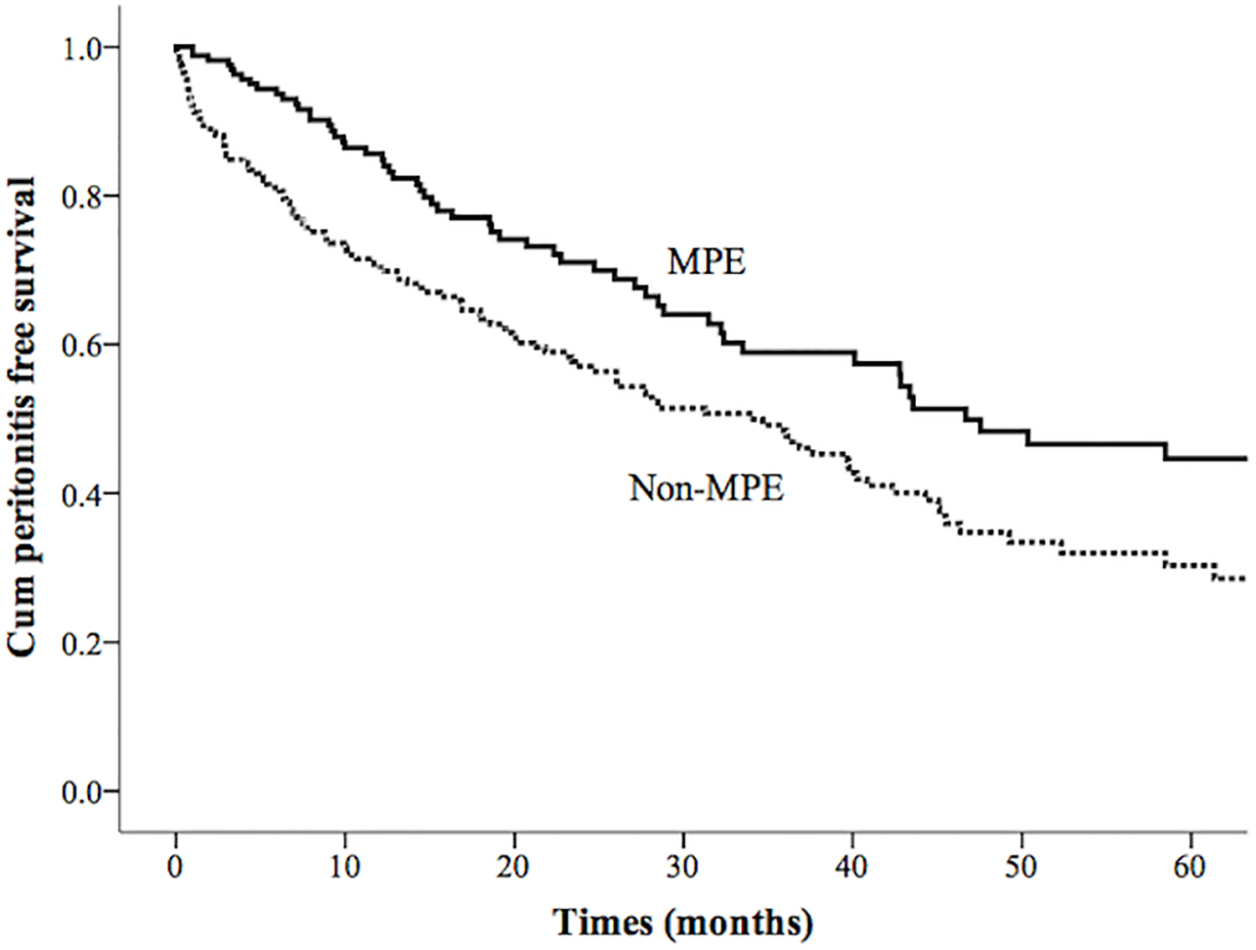
Cumulative proportion of patients who did not have peritonitis, censored for death, peritoneal dialysis dropout. Time to peritonitis was significantly longer for multidisciplinary predialysis education (MPE) recipients than for the non-recipients (non-MPE; Cox-mantel log rank test, *P* = 0.003).

The median time to the first episode of peritonitis in the non-MPE and MPE groups was 33.9 months and 46.7 months, respectively. The difference in the median time between the two groups was 12.8 months (Cox-Mantel log rank test, *P* = 0.003, Fig 2). Using Cox regression analysis, the educational level below elementary [hazard ratio (HR): 1.925; 95% confidential interval (CI): 1.257–2.874, *P* = 0.003] and the use of MPE (HR: 0.594; 95% CI: 0.434–0.813, *P*< 0.001) were significant independent predictors for peritonitis-free survival, after adjusting the baseline characteristics of age, gender, diabetes, hypertension and peritoneal modalities (Table 3).

**Table 3:** Hazard ratios for the duration from peritoneal dialysis start to the first peritonitis according to baseline characteristics.

Variables	Crude		Adjusted*	
	HR (95% CI)	*P*-value	HR (95% CI)	*P*-value
**Age**	1.017 (1.007–1.027)	0.001	1.009 (0.997–1.021)	0.12
**Male Gender**	0.840 (0.628–1.124)	0.241	0.872 (0.636–1.195)	0.40
**Diabetes (yes vs. no)**	1.104 (0.824–1.480)	0.507	1.091 (0.799–1.488)	0.58
**Hypertension (yes vs. no)**	0.952 (0.629–1.443)	0.818	0.929 (0.611–1.413)	0.73
**Eduacational status**				
Elementary and beyound	1		1	
Below elementary	2.238 (1.570–3.189)	<0.001	1.925 (1.257–2.874)	0.003
**APD (yes vs. no)**	1.102 (0.824–1.473)	0.511	1.173 (0.871–1.578)	0.29
**MPE (yes vs. no)**	0.671 (0.496–0.909)	0.010	0.594 (0.434–0.813)	<0.001

**Abbreviations**: APD, automated peritoneal dialysis; MPE, multidisciplinary predialysis education.

*Adjusted for age, gender, diabetes, hypertension, educational status, APD and MPE.

A total of 316 episodes of peritonitis and 254 microorganisms were isolated during the study period. Table 4 demonstrates the microbiologic spectrum causative of peritonitis in patients with MPE and non-MPE. Gram-positive organisms were the most common isolates of all pathogens (40.8%), followed by gram-negative organisms (36%, being the *Escherichia coli* the most common gram-negative species). Fungal organisms represented 1.9% and the *mycobacterium* species comprised 1.6% of all organisms. Higher percentage of *Streptococcus* species infection was noted in the MPE-group (23.4% versus 9.9%, *P* = 0.01). In contrast, higher percentage of culture-negative peritonitis was noted in non-MPE group (36.9% versus 13.8%, *P*< 0.001) than the patient who had received MPE. The causes of PD peritonitis due to touch and connection contamination were 62.1% in non-MPE group and 52.1% in MPE group (*P = 0*.*013*); enteric causes were 11.1% in non-MPE group and 37.2% in MPE group (*P*<0.001); causes of unknown were 26.8% in non-MPE group and 10.6 in MPE group (*P* = 0.003).

**Table 4:** Details of causative organisms of peritonitis.

	All episodes (n = 316)	Non-MPE (n = 222)	MPE (n = 94)	*P*-value
**Gram-positive organisms**	129 (40.8%)	80 (36.0%)	49 (52.1%)	0.01
*Streptococcus* species	44 (13.9%)	22 (9.9%)	22 (23.4%)	0.002
*Staphylococcus aureus*	26 (8.2%)	21 (9.5%)	5 (5.3%)	0.22
MSSA	18 (5.7%)	15 (6.8%)	3 (3.2%)	0.21
MRSA	8 (2.5%)	6 (2.7%)	2 (2.1%)	0.90
*Coagulase-negative staphylococcus*	35 (11.1%)	20 (9.0%)	15 (16.0%)	0.07
Oxacillin-sensitive	21 (6.6%)	15 (6.8%)	6 (6.4%)	0.90
Oxacillin-resistent	14 (4.4%)	5 (2.3%)	9 (9.6%)	0.007
*Enterococcus* species	18 (5.7%)	12 (5.4%)	6 (6.4%)	0.73
*Other gram-positives*	6 (1.9%)	5 (2.3%)	1 (1.1%)	0.67
**Gram-negative organisms**	114 (36.0%)	79 (35.6%)	35 (37.2%)	0.39
*Escherichia coli*	42 (13.3%)	31 (14.0%)	11 (11.7%)	0.59
*Klebsiella* species	23 (7.3%)	15 (6.8%)	8 (8.5%)	0.58
*Haemophillus* species	2 (0.6%)	1 (0.5%)	1 (1.1%)	0.53
*Pseudomonas* species	10 (3.2%)	6 (2.7%)	4 (4.3%)	0.49
*Serrati*a species	4 (1.3%)	3 (1.4%)	1 (1.1%)	0.90
*Acinetobacter* species	9 (2.8%)	6 (2.7%)	3 (3.2%)	0.73
*Other gram-negatives*	24 (7.6%)	17 (7.7%)	7 (7.4%)	0.95
**Fungi**	6 (1.9%)	3 (1.4%)	3 (3.2%)	0.37
**Mycobacterium species**	5 (1.6%)	3 (1.4%)	2 (2.1%)	0.64
**Culture negative**	95 (30.1%)	82 (36.9%)	13 (13.8%)	<0.001
**Causes of peritonitis**				
Touch and connection	187 (59.2%)	138 (62.2%)	49 (52.1%)	0.13
Enteric source	60 (19%)	25 (11.3%)	35 (37.2%)	<0.001
Unknown	69 (21.8)	59 (26.6)	10 (10.6)	0.003

**Abbreviations**: MSSA, methicillin-sensitive *Staphylococcus aureus*; MRSA, methicillin-resistent *Staphylococcus aureus*

## Discussion

In this prospective 5-years cohort study, we have demonstrated that patients who had received MPE in their predialysis period may have a lower peritonitis rate, more prolonged median time to the first episode of peritonitis from PD initiation and lower peritonitis-related mortality than non-MPE recipients. The positive effect of multidisciplinary education delivered at predialysis period may have legacy effect after starting of PD resulting in less peritonitis occurrence and subsequent death. To our knowledge, this is the first prospective long-term study, to demonstrate the beneficial effect of MPE in the outcome of PD patients. The findings of study highlight again the importance of the implementation of multidisciplinary team care as pivotal part of medical care for CKD patients.

The positive role of MPE in lowering the incidence of dialysis and reducing mortality in CKD patients has been well established [8, 14–16]. Previous studies have demonstrated that MPE was associated with better clinical outcomes of patients with advanced CKD in terms of decreased urgent dialysis and slow renal function declines [9, 14, 17, 18]. Additionally, MPE program has a significant effect on increasing the proportion of patients initiating dialysis with PD and the number of pre-emptive renal transplantations [19–22]. Furthermore, participation of MPE in predialysis period is independently associated with reduction in total medical costs of the first 6 months of dialysis in incident hemodialysis patients [11]. The apparent protective benefits of multidisciplinary “predialysis” education on patients who later start PD has been seldom discussed. One study reported that multidisciplinary management for patients after receiving PD has benefits on quality of life [23]. In this prospective cohort study, we demonstrated that the peritonitis rate was lower and the time to first episode of peritonitis was significantly longer (33.9 versus 46.7 months, a difference of 12.8 months) in MPE group than the non-recipients, although the MPE program was discontinued after PD initiation.

The beneficial legacy effects of MPE on the peritonitis rate and peritonitis-related mortality could be the results from many factors, such as selecting adequate candidates for PD during pre-dialysis period, adoption of a positive attitude towards physical fitness, empowerment of self-care technique, improvements in patient compliance with medication regimens, maintenance of a healthier lifestyle and greater awareness of the complications of PD. It is possible that this education at predialysis stage has enhanced the confidence of patient and caregiver for this self-care modality selection. It may in part explain the beneficial legacy effect of MPE at post-dialysis period. The subjectivity of MPE was minimal in this study because the program was standardized according to the NKF/DOQI guideline [12]. A single nurse instructed the MPE program limiting interpersonal variability. The MPE effectively increased self-management knowledge and it was found to be beneficial, regardless of the baseline demographic characteristics and education status [10].

Risk factors associated with the development of PD-related peritonitis include older age, diabetes and lower educational status [24–28]. However, in spite of older age, more diabetes patients and lower educational status of our MPE group, the beneficial effect of MPE on study outcome remained significant. There is a significant gender difference between the two groups of study; however, previous studies concerning gender as a risk factor of PD-peritonitis are controversial to date [25, 28], and it was unlikely to have influence in our study results.

In our study, the mortality rate per peritonitis episode was significantly lower in MPE group than non-MPE recipients (6.3% versus 9.0%, *P*< 0.001). The rate was similar to other researches reporting peritonitis-related mortality rate ranged from 4.5 to 8.4% per episode [4, 29, 30]. Short time elapsed from PD initiation to first peritonitis was a significant indicator associated with poor prognosis of patients, including higher mortality and recurrent peritonitis rates [6, 7]. Prolongation of time to first episode of peritonitis in our patients may *per se* contribute to decrease mortality. Other possible explanations to a better patient survival after peritonitis in the MPE group included increasing alerts, timely treatments for peritonitis episode resulting in less technique failure and possible less virulent microorganism than non-MPE patients. This supposition may be evident from a higher frequency of hospitalization and less shifting of modality to HD in MPE groups compared to non-MPE patients (Table 2). The predominance of *Streptococcus* species related peritonitis in MPE patients was alleviating, because of its high cure rates and low risks of PD catheter removal, especially for *Viridans streptococci* [31]. On the other hand, culture-negative peritonitis was higher in non-MPE group than MPE patients. The exact reasons for bacteriological difference between the two groups were peculiar. The percentage of erroneous touch or connection causing peritonitis was similar between the both group. However, MPE patients had greater proportion of enteric source of peritonitis than non-MPE patients. Care plan to enhance enteric health should be further implemented as content of multidisciplinary education for CKD patients.

There are several limitations concerning to our study. First, assignment to MPE program was not at random before starting PD. All CKD patients who attended our outpatient clinics were instructed to receive MPE. However, the MPE team was available during habitual working days with fixed schedule. Patients who expressed difficulty to adhere the schedule (for example, patients of nocturnal or weekend clinics) and had irregular participation on education program were considered as non-MPE patients. It is possible that young patients playing roles in employment or those patients with less disease severity perception (non-diabetics) represented the most patients of non-MPE group. This supposition might be partially supported by the baseline feature of patients started in Table 1. It was less likely that possible patient diversity from baseline have influence in the study outcome. Second, we did not measure the self-care knowledge score for PD treatment in each patient of both groups. Furthermore, the causal relationship between better empowerment of self-care technique of PD and better outcome of MPE patients could not be inferred. However, patients of the two groups were instructed by the same PD nursing and nephrology physician staff. Introduction of PD technique was standardized using informative teaching materials and booklet to ensure proper home-based care. Other limitations of generalizability were found, including different ethnic groups, single-center experience, lack of information regarding socioeconomic condition of patients. However, the prospective design, extensive follow-up period and coverage of important clinical risk factor have strengthened the conjecture of this study.

In conclusion, an efficient standardized MPE program adhered to the NFK/DOQI guidelines may prolong the time to the first episode of peritonitis and reduce peritonitis rate, independent of age, gender, diabetes, hypertension, educational status and PD modality. Subsequently, decreased peritonitis-related death. The findings provided basis for strategic implementation of MPE as an efficient method to improve dismal outcome of PD patients.

## Supporting information

S1 File Raw data used for this manuscript. (XLSX)
